# Successful management of life-threatening acute pulmonary embolism during anesthesia induction for comminuted intertrochanteric fracture surgery: a case report

**DOI:** 10.3389/fcvm.2026.1791835

**Published:** 2026-06-22

**Authors:** Minghao Tang, Jing Zhao, Ruijun Tong

**Affiliations:** Department of Anesthesiology, The First People’s Hospital of Jiashan, Jiaxing, Zhejiang Province, China

**Keywords:** acute pulmonary embolism, anticoagulation therapy, arterial blood-gas analysis, case report, fracture surgery, thrombolysis

## Abstract

**Background:**

Acute perioperative pulmonary embolism (APE) is a life-threatening complication that demands immediate clinical recognition and intervention. This report describes the emergency management and successful recovery of an 85-year-old female who experienced sudden, catastrophic cardiovascular collapse due to APE immediately following general anesthesia induction for orthopedic surgery.

**Methods:**

A retrospective analysis was conducted on an 85-year-old female admitted with an un-stabilized comminuted intertrochanteric fracture of the right femur. The study chronologically details the clinical presentation during an 11-day preoperative immobilization window, the intraoperative crisis management utilizing real-time bedside diagnostics, and the subsequent multidisciplinary rescue protocol involving targeted pharmacological thrombolysis and intensive care stabilization.

**Results:**

Initial screening revealed a profound hypercoagulable state. Following anesthesia induction, the patient experienced sudden cardiovascular collapse and new-onset atrial fibrillation with complete right bundle branch block. Bedside echocardiography rapidly confirmed right ventricular enlargement, indicating massive APE and obstructive shock. An emergency low-dose systemic thrombolysis regimen (50 mg alteplase) was successfully executed. Post-thrombolysis evaluations in the intensive care unit noted expected consumptive coagulopathy and transient anemia, managed via supportive care and blood transfusion. Computed tomography pulmonary angiography confirmed bi-lobed pulmonary embolism. Under multidisciplinary optimization and transitioned rivaroxaban therapy, the patient achieved complete clinical recovery at 1-year follow-up.

**Conclusions:**

This case underscores the importance of intraoperative point-of-care ultrasound and electrocardiographic monitoring in fragile geriatric patients undergoing extended preoperative delays. When mechanical options are unavailable, low-dose systemic thrombolysis can serve as a viable rescue pathway to reverse acute obstructive shock, though its wider generalizability requires cautious interpretation.

## Introduction

Acute perioperative pulmonary embolism (APE) stands as one of the most catastrophic complications encountered by perioperative teams (1, 2). In elderly patients sustaining major hip or lower-extremity fractures, the mandatory window of preoperative immobilization creates a highly volatile pathophysiological breeding ground for venous thromboembolism (VTE) (3). Recent clinical evidence clarifies that advanced age (>60 years) operates as a powerful independent risk factor for preoperative deep vein thrombosis (DVT), driven by an aggressive baseline hypercoagulable state characterized by markedly elevated fibrinogen levels, suppressed antithrombin III activity, and acute-phase systemic inflammation (4, 5). When these skeletal injuries are accompanied by chronic systemic comorbidities—such as hypertension, diabetes, or chronic kidney disease—the susceptibility to profound endothelial damage and microcirculatory stagnation increases exponentially (3).

Furthermore, multi-center risk-stratification models emphasize that a prolonged delay from initial skeletal trauma to surgical fixation severely worsens this prothrombotic drive (3, 6). Over dates of mandatory immobilization, the local and systemic activation of immune-mediated coagulation pathways—often quantified via elevated neutrophil-to-lymphocyte ratios (NLR) and D-dimer matrices—builds an incredibly stubborn clot bulk within the deep venous network (3, 6, 7). Despite standard low-molecular-weight heparin (LMWH) thromboprophylaxis and even false-negative baseline Doppler compression ultrasounds, this hypercoagulable momentum remains aggressively active, maintaining a silent, lethal stage for massive embolic detachment during early operative maneuvers or anesthetic positioning (3).

Crucially, when massive APE manifests abruptly during anesthesia induction, its clinical footprint heavily mimics other high-mortality entities, such as severe anaphylactic shock or acute myocardial infarction, making rapid bedside differentiation exceptionally challenging (8). Furthermore, when obstructive circulatory collapse occurs prior to surgical fixation, the decision to initiate emergency systemic chemical thrombolysis represents a profound clinical dilemma, as the immediate life-saving benefits of myocardial reperfusion must be aggressively weighed against the high risk of catastrophic hemorrhage at the un-stabilized fracture site (9).

Herein, we present the successful multidisciplinary resuscitation of an 85-year-old female with an un-stabilized intertrochanteric fracture who suffered sudden intraoperative massive APE and obstructive shock immediately following anesthesia induction and position adjustment. This report specifically analyzes her highly tumultuous preoperative warning signs rooted in recent VTE risk-stratification frameworks, the rapid intraoperative diagnostic clues that directed the rescue, the explicit rationale behind our emergency low-dose alteplase protocol, and actionable perioperative learning points for acute crisis management in fragile geriatric cohorts.

## Case report

### Chief complaint

An 85-year-old female presented with a one-day history of persistent pain, swelling, and functional impairment of the right femur due to a traumatic fall. She was received at The First People's Hospital of Jiashan (Jiashan Branch of the Second Affiliated Hospital of Zhejiang University School of Medicine) on August 15, 2024.

### History of past illness

Her past medical history was notable for essential hypertension, diabetes, and atrial fibrillation, for which she had been receiving chronic medical therapy (specific agents unspecified).

### Family history

The patient's family history was unremarkable. There was no history of similar clinical conditions among family members. Immediate family members were reported to be in good health. There was no family history of infectious diseases, malignant tumors, or genetic disorders within three generations of the patient's lineage.

### Physical examination

On admission, vital signs were stable (T: 36.8 °C, P: 76 bpm, R: 19 breaths/min, BP: 104/78 mmHg). The patient was conscious (VAS pain score: 4/10) with no remarkable findings in the skin, lymph nodes, or cardiopulmonary and abdominal examinations. Neurological status was intact with negative pathological reflexes. The spine showed normal alignment and mobility. The right hip was significantly swollen with localized tenderness. The right lower limb presented a characteristic shortening and external rotation deformity with severely restricted range of motion (ROM) due to pain. Distal neurovascular status, including toe motor-sensory function and capillary refill, remained intact.

### Laboratory examinations & imaging examinations (At admission)

Laboratory investigations upon admission (Admission phase) revealed significant cardiac and metabolic stress, characterized by an elevated B-type Natriuretic Peptide (BNP) of 385.9 pg/mL and cardiac Troponin I (cTnI) of 0.053 ng/mL, alongside a compensated metabolic acidosis (pH: 7.358; PaCO2: 28.0 mmHg; Lactate: 2.1 mmol/L) (Table 1). Consequently, the patient was preliminarily diagnosed with a right-sided comminuted intertrochanteric fracture, essential hypertension, type 2 diabetes mellitus, chronic atrial fibrillation, chronic heart failure, cholecystitis with cholelithiasis, and a right renal cyst. At admission, the patient's D-dimer was already significantly elevated at 18,330 μg/L, while other parameters like Fibrinogen (FIB: 2.100 g/L) and INR (1.07) remained within baseline reference ranges (Table 1).

**Table 1:** Coagulation function test.

Parameters	Admission (Aug 15)	Pre-op Max (Aug 23)	Intra-op Crisis (Aug 26, 10:54)	Post-Thrombolysis Peak (Aug 26, ICU)	Post-Crisis Recovery (Aug 29)	Pre-Discharge Assessment (Sep 03)	Follow-up (next year, Nov 05)	Ref.
D-dimer (*μ*g/L)	18,330	20, 070	39,080	116,000	12,680	39,030	1,350	0–550^a^
INR	1.07	1.03	1.10	1.47	1.04	1.01	0.94	0.90–1.20
APTT (s)	21.2	26.3	30.0	57.6	30.6	37.3	26.5	23.5–36.0
PT (s)	12.8	12.4	13.2	17.4	12.5	12.1	11.3	
FIB (g/L)	2.100	3.174	3.010	0.390	1.755	4.701	2.521	2.000–4.000
TT (s)	18.8	16.9	18.1	31.1	20.5	17.2	17.6	15.0–23.0

INR, international normalized ratio; APTT, activated partial thromboplastin time; PT, prothrombin time; FIB, fibrinogen; TT, thrombin time.

a 0–550 normal, 550–1800 low risk,1800–3600 medium risk,3600–6600 high risk.

**Figure 1:**
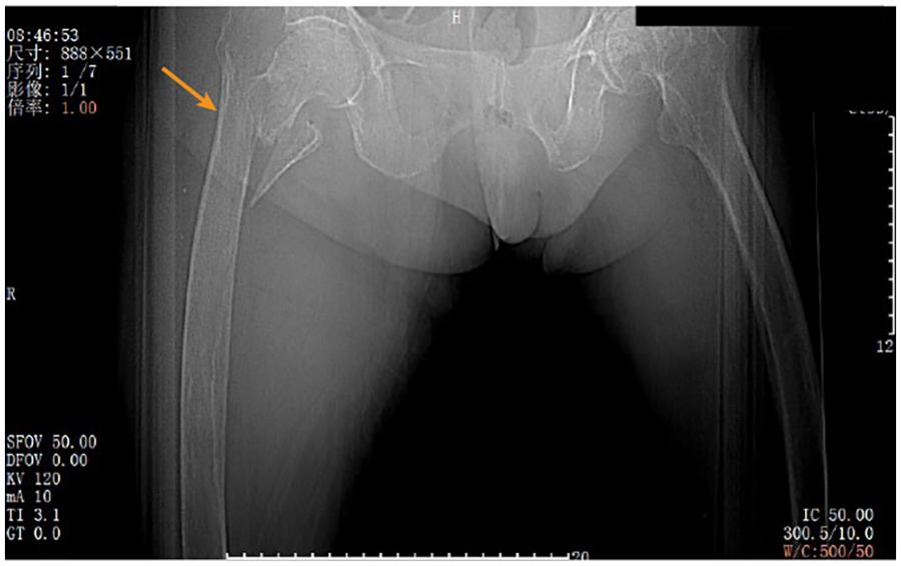
Digital radiograph (DR) revealing the right comminuted intertrochanteric fracture performed on August 15, 2024.

Preoperative imaging performed on August 15, including digital radiography (DR) and multi-region CT scans, confirmed a comminuted intertrochanteric fracture of the right femur (Figure 1). Cranial CT ruled out acute intracranial hemorrhage, noting only age-related cerebral changes and a localized encephalomalacia focus. While chest and abdominal CT scans identified chronic conditions such as lingular inflammation, cholelithiasis, and a renal cyst, no acute visceral hemorrhage was detected. Notably, a complete lower-extremity Doppler ultrasound was performed on August 19, which explicitly reported completely patent deep veins and no deep vein thrombosis (DVT) (Supplementary Figure 1). Additionally, preoperative echocardiography demonstrated an enlarged left atrium (LA: 44 mm) with preserved systolic function (EF: 70%), consistent with the patient's history of chronic atrial fibrillation (Supplementary Figure 2).

### Preoperative management and multidisciplinary optimization

Owing to her advanced age (85 years) and multi-organ frailty, the patient's preoperative course was complex, necessitating an 11-day window of intensive optimization (Table 2). On admission (August 15), baseline testing demonstrated prominent systemic distress, including a high thromboembolic liability (CHA2DS2-VASc score of 4) secondary to chronic atrial fibrillation, which had been restricted to long-term antiplatelet therapy (aspirin) rather than therapeutic anticoagulation, alongside chronic heart failure (NYHA Class II) and compensated metabolic acidosis (Tables 1, 2). Following a multidisciplinary consultation on August 16, the home use aspirin was immediately discontinued to mitigate perioperative bleeding risks, and bridging thromboprophylaxis was initiated with standard low-molecular-weight heparin (LMWH), supplemented by passive lower-limb physical exercises (Table 2). Concurrently, targeted heart failure optimization was enforced using oral furosemide (10 mg, qd), spironolactone (20 mg, qd), and potassium chloride (0.5 g, bid) (Table 2).

**Table 2:** Chronological summary of targeted pharmacological interventions and clinical rationale.

Phase/Time	Identified Clinical Status & Challenge	Administered Agent(s) & Dosage
Preoperative Phase(Aug 15 – Aug 25)	An 85-year-old female with an intertrochanteric fracture and multiple comorbidities (AF, CHF, CRI, HTN, T2DM) presented with marked clinical instability, characterized by a profound hypercoagulable state (D-dimer: 20,070 μg/L) and recurrent myocardial injury (cTnI: 0.339 ng/mL). Concurrently, bilateral pleural effusion, moderate anemia (Hb: 87 g/L), and hypoalbuminemia (ALB: 28.6 g/L) were noted.	• VTE Prophylaxis: Routine chemical anticoagulation via low-molecular-weight heparin (LMWH) was actively maintained from Aug 16 onward, supplemented by regular physical lower-limb exercises to prevent deep vein thrombosis (DVT). • Cardiorenal & Supportive Optimization: Discontinuation of Aspirin; administration of Isosorbide Mononitrate (20 mg bid), Atorvastatin (20 mg qn), Furosemide, Spironolactone, and Iron Sucrose for anemia correction.
Aug 26, 10:54(Intra-op Crisis)	Sudden hypotension and hypoxia after general anesthesia (before surgery). Bedside Echo: enlarged RA and RV with moderate tricuspid regurgitation. ECG: AF with rapid ventricular rate, CRBBB. D-dimer: 39,080 μg/L.	Emergency Thrombolysis & ICU Transfer: Immediate intravenous thrombolysis with Alteplase (50 mg, single dose) was executed. Patient was transferred to the ICU under endotracheal intubation and mechanical ventilation.
Aug 26, ICU Stay(Post-Thrombolysis Peak)	Deep coma. Extreme thrombolysis-induced coagulopathy: FIB dropped to 0.390 g/L (Critical Value); D-dimer peaked at 116,000 μg/L. Progressive myocardial injury: cTnI rose to 0.244 ng/mL, peaking at 2.163 ng/mL overnight. Severe metabolic acidosis (pH 7.118, Lactate 3.8 mmol/L). Severe hypoalbuminemia.	Dual vasoactive support (Norepinephrine + Pituitrin) to maintain blood pressure. Prophylactic broad-spectrum antibiotics (Piperacillin/Tazobactam 4.5 g iv q8h). Intensive organ protection: myocardial nutrition (Phosphocreatine), neuro-protection (Xingnaojing), and PPIs. Human albumin supplementation (20 g) infused.
Aug 27(Anticoagulation Strategy)	Severe fluid positive balance (Intake: 5236 mL, Output: 1835 mL). Persistent low fibrinogen (FIB: 0.733 g/L) but no active hemorrhage. cTnI peaked at 3.024 ng/mL (low-oxygen induced myocardial damage). Severe stress-induced hyperglycemia.	Introduction of Anticoagulation & Heart Failure Management: Dalteparin sodium (0.2 mL ih qd) was carefully initiated post-thrombolysis. Digoxin (0.5 tab qd) and intravenous Torasemide were added for CHF and oliguria. Hyperglycemia was managed via continuous insulin infusion and oral Metformin. Combined enteral and parenteral nutrition support established.
Aug 28(Definitive Diagnosis & Weaning)	Pulmonary CTA confirmed bi-lobed pulmonary embolism with bilateral pleural effusion and compressive atelectasis. Neurological status improved to somnolence (complying with simple commands). Hemodynamics stabilized with tapered vasoactive drugs.	Anticoagulation Intensification & Extubation: Dalteparin sodium was up-titrated to full-dose therapeutic anticoagulation (0.2 mL ih q12h). Mechanical ventilation was successfully weaned, and the endotracheal tube was extubated to face mask oxygen. Diuresis escalated (Torasemide increased) and Ginkgo Damo added to improve microcirculation.
Aug 29–30(Stabilization & Ward Transfer)	Patient fully conscious and stable on nasal cannula oxygen. Negative fluid balance achieved (Aug 29 Output: 3400 mL vs. Intake: 2479 mL). Blood gas revealed severe anemia (Hb: 7.7 g/dL). Secondary renal impairment noted (Cr: 163 μmol/L).	Anemia Correction & Orthopedic Ward Transfer: Blood transfusion of 400 mL (2.0 U) of leukocyte-depleted suspended red blood cells was successfully administered on Aug 30. Diuretics were tapered following nephrology consultation. Patient was safely transferred back to the orthopedic ward at 14:00 on Aug 30 for further surgical planning.
Discharge & Follow-up (next year, Nov 05)	• Patient fully recovered with stable vital signs; follow-up echocardiogram showed a well-preserved EF of 70%, and lower extremity venous duplex ultrasound revealed no residual DVT. •Surgical incision healed properly; deemed fit for discharge.	• Oral anticoagulation: Rivaroxaban (10 mg qd, PO). • Routine post-orthopedic follow-up and periodic coagulation profile monitoring.

A major diagnostic and management dilemma unfolded between August 17 and August 23. On August 17, a critical value notification was triggered as cTnI abruptly surged to 0.339 ng/mL. Notably, the patient experienced no obvious chest pain or typical ischemic symptoms, presenting only with her baseline arrhythmia. Given the intense physiological stress of the skeletal trauma in this frail patient, this subacute myocardial injury was clinically interpreted and managed as a suspected Type II myocardial infarction secondary to supply-demand mismatch. Immediate protective measures, including continuous ECG monitoring and oxygen therapy, were implemented. On August 19, a follow-up test showed a persistently elevated cTnI of 0.200 ng/mL, prompting an urgent cardiology re-consultation. The diagnosis of coronary artery disease was corroborated, and her regimen was further optimized with oral isosorbide mononitrate (20 mg, bid) and atorvastatin (20 mg, qn) alongside ongoing LMWH anticoagulation. To correct her moderate anemia, intravenous iron sucrose was supplemented on August 18 (hemoglobin: 87 g/L) (Table 2).

As the clinical picture evolved, the patient developed worsening dyspnea and progressive renal insufficiency. Subsequent evaluations on August 20 and 21 by the nephrology and pulmonology departments identified bilateral pleural effusion, which was managed conservatively with oral febuxostat, sodium bicarbonate, and strict fluid balance monitoring. By August 23, although her cTnI had successfully declined to 0.047 ng/mL following tailored cardiopulmonary optimization, a subsequent laboratory evaluation revealed a profoundly elevated D-dimer of 20,070 μg/L (Table 1). This marked D-dimer spike, paired with the negative bilateral lower-limb Doppler ultrasound results from August 19 (confirming completely patent deep veins), strongly pointed away from deep vein thrombosis propagation and suggested an occult primary thrombotic process or *in-situ* pulmonary thrombosis. On August 25, following a comprehensive multidisciplinary pre-operative discussion, the patient's cardiopulmonary reserve and metabolic profile were deemed stabilized, and she successfully proceeded to right femoral intertrochanteric fracture reduction and intramedullary nailing.

### Sudden crisis during anesthesia explicit thrombolysis rationale and technique

At approximately 10:54 AM, following the induction of general anesthesia but prior to the surgical incision, the patient experienced a sudden drop in arterial blood pressure and oxygen saturation. An emergency multidisciplinary team (MDT) consultation—comprising directors from Cardiology, Pulmonology, the Ultrasound Department, the ECG Room, the Intensive Care Unit (ICU), and the Medical Affairs Bureau—was immediately convened to assist in the treatment. Bedside ultrasonography demonstrated enlargement of both the right atrium and right ventricle, while the ECG revealed atrial fibrillation with a rapid ventricular rate, a complete right bundle branch block (CRBBB), and a right-axis deviation.

Considering the high probability of a pulmonary embolism, emergency systemic thrombolysis was initiated with a single dose of Alteplase (50 mg via micro-pump), after which the patient was transferred to our department for further intensive monitoring and management. A crisis coagulation profile drawn at 10:54 AM confirmed massive fibrin clot lysis, with D-dimer jumping to an extraordinary level of 39,080 μg/L. The therapeutic response was immediate and profound (Table 2).

### ICU stabilization and post-thrombolysis hemorrhagic assessment

Upon transfer to the ICU at 13:48 PM on August 26 (Post-Thrombolysis Peak phase), the patient presented in a deep coma (Table 2). Laboratory evaluations exposed a profound, expected post-thrombolytic consumptive coagulopathy: Fibrinogen (FIB) dropped to a critical nadir of 0.390 g/L, Activated Partial Thromboplastin Time (APTT) was prolonged to 57.6 s, and D-dimer reached an extreme peak of 116,000 μg/L (Table 1). Concurrently, the post-thrombolysis blood gas analysis revealed severe metabolic acidosis with a pH of 7.118 and an elevated lactate of 3.8 mmol/L (Table 1). This was accompanied by severe myocardial ischemia secondary to the obstructive shock, with Troponin I rising to a peak value of 2.163 ng/mL overnight (eventually reaching a maximum of 3.024 ng/mL on August 27), while NT-proBNP rose to 503.2 pg/mL (Table 1).

**Figure 2:**
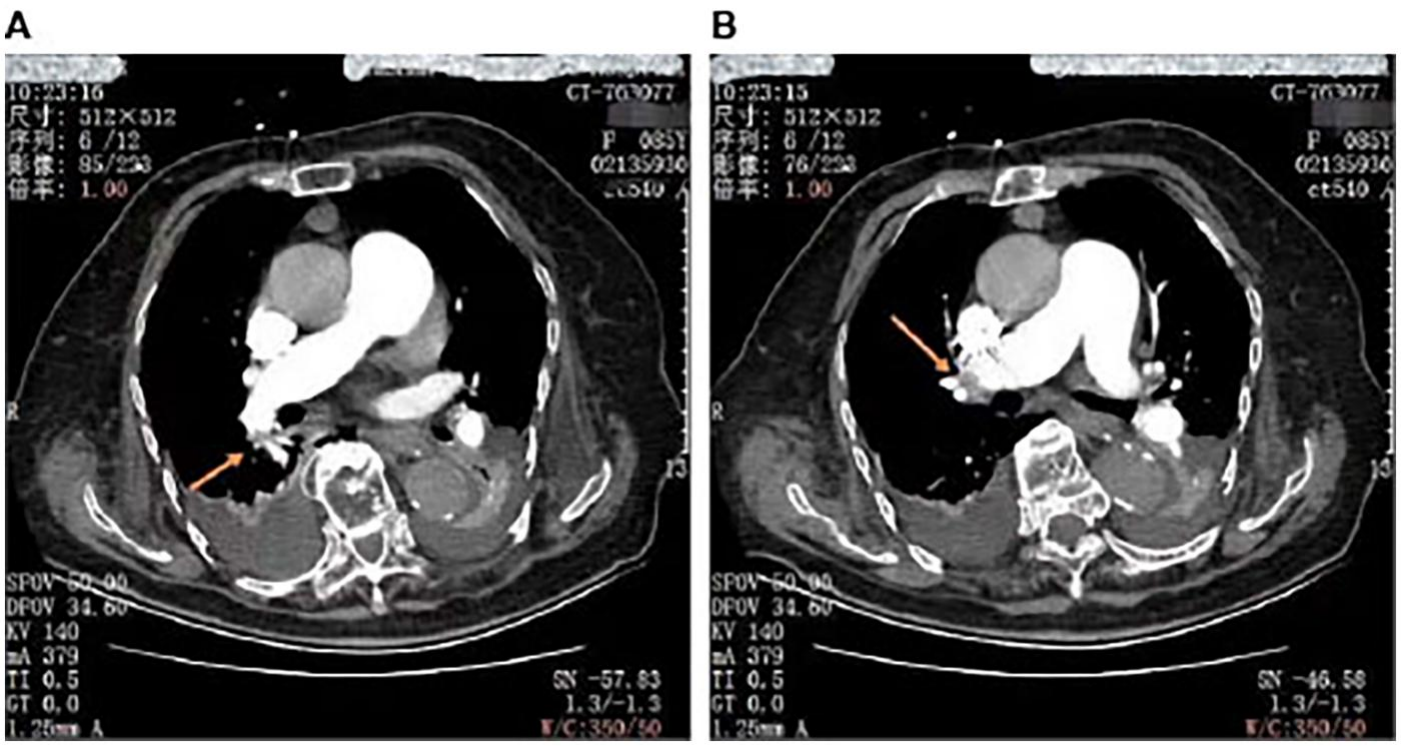
Computed tomography pulmonary angiogram (CTPA) revealing pulmonary embolism in the right upper and lower lobes, associated with bilateral pleural effusion and dependent atelectasis (August 28, 2024). **A**. Arrow indicates: Axial view of pulmonary embolism in the right lower lobe pulmonary artery. **B**. Arrow indicates: Axial view of pulmonary embolism in the right main pulmonary artery.

To sustain organ perfusion while protecting against re-thrombosis, advanced vasopressors (Norepinephrine and Pituitrin) were coupled with low-molecular-weight heparin (Dalteparin sodium, 0.2 mL ih qd) as soon as the rt-PA infusion concluded (Table 2). A definitive Pulmonary CTA (CTPA) performed on August 28 successfully confirmed the diagnosis of bi-lobed pulmonary embolism with bilateral pleural effusion and compressive atelectasis (Figure 2). Following this confirmation and ensuring no active hemorrhage, Dalteparin sodium was safely up-titrated to a full-dose therapeutic regimen (0.2 mL ih q12h) on August 28 (Table 2). Crucially, despite the volatile combination of an open fracture site and full-dose chemical anticoagulation, no major visceral or intracranial bleeding complications occurred. Minor, controlled incisional oozing from the right lower limb skeletal traction site was effectively managed with local pressure dressing. However, due to severe consumptive anemia and hemodilution, the patient's hemoglobin plummeted to a nadir of 77 g/L by August 29–30 (Table 2). To correct this anemia and optimize her for subsequent surgery, a blood transfusion of 400 mL (2.0 U) of leukocyte-depleted suspended red blood cells was successfully administered on August 30 (Table 2). By August 28, the patient was successfully weaned from mechanical ventilation and extubated (Table 2).

Following initial stabilization, sequential multi-system objective examinations were scheduled to evaluate her cardiovascular recovery and rule out residual embolic sources. On August 29 (Post-Crisis Recovery phase), a follow-up bedside echocardiography demonstrated a significantly relieved right ventricular overload with only mild pulmonary hypertension; concurrently, her systemic metabolic status and core coagulation parameters had gradually stabilized (pH: 7.471; Lactate: 2.1 mmol/L; FIB: 1.755 g/L (Table 3); D-dimer: 12,680 μg/L; as detailed in Table 1). At 14:00 on August 30, she was safely transferred back to the orthopedic ward for further surgical planning (Table 2). Subsequently, on September 3 (Pre-Discharge Assessment phase), a crucial follow-up lower-extremity Doppler ultrasonography re-confirmed persistent patency in the bilateral deep veins with no residual or recurrent macro-thrombi. Concurrently, a follow-up non-contrast cranial and chest CT scan on September 3 ruled out delayed intracranial hemorrhage and confirmed resolving pulmonary parenchymal changes, further verifying the late-stage safety of our emergency reduced-dose thrombolysis and continuous anticoagulant framework.

**Table 3:** Cardiac biomarkers, inflammatory profiles and metabolic and respiratory profiles.

Parameters	Admission (Aug 15)	Pre-op Max (Aug17- 23)	Post-Thrombolysis Peak (Aug 26, ICU)	Post-Crisis Recovery (Aug 29)	Pre-Discharge Assessment (Sep 03)	Follow-up (next year, Nov 05)	Ref.
Troponin I (ng/mL)	0.053	0.339	2.163	-	-	0.046	0.000–0.034
BNP/NT-proBNP (pg/mL)	385.9	-	503.2	433.1	615.9	104.8	0.0–100.0
LDH (U/L)	262	-	1312	-	326	233	120–250
AST (U/L)	35	-	199	32	18	27	13–35
WBC (10^9/L)	14.8	7.8	20.9	11.8	7.6	-	3.5–9.5
NEUT% (10^9/L)	91.6	82.5	87.9	87.2	83.2	-	40.0–75.0
hs-CRP (mg/L)	1.91	43.69	9.69	13.22	79.54	-	0.00–8.00
pH	7.358	7.423	7.118	7.471	7.500		7.350–7.450
P_ET_CO_2_ (mmHg)	28.0	32.0	61.5	32.1	31.1		35.0–45.0
Lactate (mmol/L)	2.1	1.2	3.8	2.1	1.6		0.5–1.6

Troponin I, cardiac troponin I; BNP, brain natriuretic peptide; NT-proBNP, N-terminal pro-B-type natriuretic peptide; LDH, lactate dehydrogenase; AST, aspartate aminotransferase; WBC, white blood cell; NEUT%, neutrophil percentage; hs-CRP, high-sensitivity C-reactive protein; P_ET_CO_2_, end-tidal carbon dioxide pressure; Ref., reference interval. Hyphens (–) indicate that no measurement was performed at that specific time point.

### Long-term follow-up

The patient recovered successfully and was discharged on September 8, 2024, on oral Rivaroxaban (10 mg, once daily) to ensure long-term secondary VTE prevention (Table 2). At her 1-year follow-up on November 05, 2025, she demonstrated complete clinical recovery; her D-dimer had safely resolved to 1,350 μg/L, and her complete coagulation and metabolic matrices had returned entirely to baseline reference limits (Table 1).

## Discussion

The occurrence of a near-fatal APE despite standard guidelines-compliant thromboprophylaxis exposes a critical perioperative blind spot in fragile trauma patients (10, 11). Upon admission (Admission phase), this patient's D-dimer was already profoundly elevated at 18,330 μg/L, further escalating to a preoperative maximum of 20,070 μg/L (Pre-op Max phase) during her mandatory 11-day immobilization. While bridging prophylactic LMWH was correctly initiated following the discontinuation of chronic home-use aspirin (3, 5), a pivotal, yet easily misinterpreted, warning sign occurred early in the admission phase on August 17, when her cardiac troponin I (cTnI) abruptly surged to a critical value of 0.339 ng/mL. In retrospect, this persistent myocardial injury was a silent, advanced herald of right ventricular (RV) strain caused by subclinical, progressive micro-thromboembolic propagation prior to overt hemodynamic collapse (12, 13). Crucially, the lower-extremity Doppler ultrasound performed on August 19 reported completely patent deep veins, which seemingly validated the clinical assumption of isolated cardiac distress and provided a false sense of security. This distinct presentation underlines that while skeletal trauma and physiological stress frequently induce a transient Type II myocardial infarction via supply-demand mismatch (14, 15), an unyielding, out-of-proportion cTnI elevation in the presence of profound thrombo-inflammatory activity (D-dimer >18,000 μg/L) serves as a critical proxy for subclinical pulmonary vascular bed occlusion (16). This clinical course highlights a dangerous diagnostic pitfall: negative lower-extremity imaging must never eliminate the suspicion of an evolving thromboembolic process when prolonged immobilization is coupled with unyielding biomonitoring derangements (6, 7, 17–19).

The catastrophic spike of D-dimer to 20,070 μg/L on August 23—occurring days *after* a clean lower-limb ultrasound—strongly implies that the eventual APE did not originate from a classic migrating deep vein thrombosis. This phenomenon aligns with growing evidence from severe trauma cohorts, where isolated PE without accompanied DVT is increasingly recognized as a distinct clinical entity. As documented by similar trauma registry data, post-traumatic PE frequently occurs independently of lower-extremity venous thrombosis, driven by unique injury-related hypercoagulability and localized thrombo-inflammatory pathways (20). Consequently, the persistently negative lower-extremity ultrasound results both preoperatively and post-embolism in our patient strongly substantiate that the APE was driven by an occult primary thrombotic process within the pelvic plexuses or directly via an *in-situ* pulmonary artery thrombosis fueled by a localized thrombo-inflammatory storm.

Sudden hemodynamic collapse following general anesthesia induction carries a near 100% imminent mortality risk, requiring immediate, systematic intraoperative differentiation (12). Anesthetic anaphylaxis or primary myocardial depression can induce profound shock, but they fail to explain the concurrent right-axis deviation and metabolic crisis (pH: 7.118, Lactate: 3.8 mmol/L) (Table 1). While localized coronary ischemia was suspected due to her frailty, the ECG demonstrated a new-onset complete right bundle branch block (CRBBB) rather than left ventricular ST-segment changes. Furthermore, fat embolism syndrome (FES) is strictly linked to intramedullary reaming or cementation (21). This patient's collapse occurred during the quiet phase of anesthesia induction prior to any surgical incision, making FES highly improbable. Consequently, the sudden onset of atrial fibrillation with a rapid ventricular response, a new-onset CRBBB, and profound hypotension pointed directly toward acute RV outflow tract obstruction. This mechanical signature was rapidly confirmed via point-of-care bedside transthoracic echocardiography (TTE), which demonstrated acute cor pulmonale characterized by prominent right atrial and right ventricular chamber enlargement, providing the definitive diagnostic justification to prioritize APE over other confounding entities (22).

Initiating emergency chemical thrombolysis in an 85-year-old patient with an un-stabilized, highly vascular intertrochanteric fracture represents a perilous risk-benefit balance, as fibrinolytics carry a near-prohibitive risk of catastrophic hemorrhage at the acute trauma site. However, facing impending circulatory arrest and lacking mechanical reperfusion alternatives like ECMO or mechanical thrombectomy, immediate right ventricular unloading immeasurably outweighed localized bleeding liabilities, a salvage strategy heavily supported by emergency literature (23). To minimize systemic hemorrhagic risks, a reduced-dose alteplase regimen (50 mg total) was executed, validating the clinical safety framework of the landmark PEITHO-3 trial (24) by successfully restoring hemodynamic stability without inducing major visceral or intracranial bleeding. Notably, the secondary D-dimer rebound from 12,680 μg/L on August 29–39,030 μg/L on September 3 represented a benign, multi-factorial phenomenon driven by accelerated continuous lysis of residual micro-clots under therapeutic anticoagulation combined with the profound systemic fibrinolytic stress of subsequent definitive internal fixation. This interpretation was robustly validated by complete bilateral deep vein patency on follow-up Doppler ultrasound, resolving pulmonary changes on chest CT, and absolute clinical stability throughout the perioperative window. Recognizing this multi-factorial biomarker rebound is clinically vital to prevent unnecessary diagnostic panic or inappropriate alterations to an already successful antithrombotic strategy.

This complex case yields vital, actionable insights that align closely with the principles delineated in the latest 2026 AHA/ACC/ACCP/CHEST Guideline for APE (12). First, a negative baseline lower-extremity venous ultrasound should never be utilized to dismiss the threat of APE in patients undergoing extended preoperative immobilization with hypercoagulable biomarkers. Second, anesthesia and perioperative teams must maintain a low threshold for utilizing intraoperative point-of-care TTE. The sudden onset of tachyarrhythmia combined with a new-onset CRBBB and hypotension must be managed as acute massive APE until proven otherwise. Third, regional centers lacking mechanical thrombectomy or advanced ECMO capabilities must maintain immediate availability of low-dose rt-PA protocols (50 mg). The narrow therapeutic window to reverse acute obstructive shock does not permit delayed inter-hospital transfer. Finally, following successful emergency fibrinolysis, transitioning to a tailored, prophylactic-to-therapeutic direct oral anticoagulant regimen (such as Rivaroxaban) provides highly effective long-term secondary prevention while minimizing bleeding in frail, elderly patients with remaining musculoskeletal trauma.

## Conclusion

In conclusion, this case demonstrates that acute perioperative pulmonary embolism during the induction of general anesthesia can present as an immediate, life-threatening crisis that necessitates rapid clinical recognition. While extreme preoperative biomarker elevations like D-dimer signal an aggressive underlying hypercoagulable state and serve as critical indicators of subacute risk, the definitive intraoperative decision to initiate emergency intervention must be guided primarily by sudden hemodynamic collapse, characteristic electrocardiographic shifts, and immediate bedside echocardiographic evidence of right ventricular strain, rather than awaiting laboratory confirmation. Furthermore, when advanced mechanical thrombectomy or extracorporeal circulatory support is unavailable at regional centers, the cautious utilization of an emergency low-dose systemic thrombolytic protocol may offer a viable rescue pathway to reverse obstructive shock, even in high-risk geriatric cohorts with un-stabilized skeletal trauma. However, because these observations are derived from a single clinical experience, the therapeutic safety, optimal dosing regimens, and definitive outcomes cannot be broadly generalized into standard practice guidelines. Further extensive controlled trials and multi-center registries are required to validate the wider applicability and safety margins of reduced-dose fibrinolytic strategies in fragile surgical patients.

## Data Availability

The original contributions presented in the study are included in the article/Supplementary Material, further inquiries can be directed to the corresponding author.

## Supplementary material

Supplementary Figure 1 Preoperative lower-extremity color Doppler ultrasonography performed on August 19.

Supplementary Figure 2 Preoperative transthoracic echocardiography showing structural left atrial alterations.
