# Supplementary figures and images for: Successful management of life-threatening acute pulmonary embolism during anesthesia induction for comminuted intertrochanteric fracture surgery: a case report
Source: Front Cardiovasc Med. 2026 Jun 22;13:1791835. doi: 10.3389/fcvm.2026.1791835 (PMC13333718; doi:10.3389/fcvm.2026.1791835)

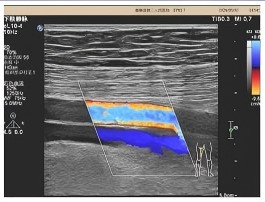

Supplement: Supplementary Figure 1 — Preoperative lower-extremity color Doppler ultrasonography performed on August 19. [file Image1.jpeg]

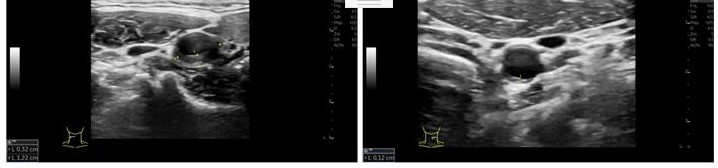

Supplement: Supplementary Figure 2 — Preoperative transthoracic echocardiography showing structural left atrial alterations. [file Image2.jpeg]
